# The Phenoxyphenol Compound 4-HPPP Selectively Induces Antiproliferation Effects and Apoptosis in Human Lung Cancer Cells through Aneupolyploidization and ATR DNA Repair Signaling

**DOI:** 10.1155/2020/5167292

**Published:** 2020-01-07

**Authors:** Wangta Liu, Chang-Yi Wu, Mei-Jei Lu, Yung-Jen Chuang, Eing-Mei Tsai, Steve Leu, I-Ling Lin, Chih-Jan Ko, Chien-Chih Chiu, Wen-Tsan Chang

**Affiliations:** ^1^Department of Biotechnology, Kaohsiung Medical University, Kaohsiung 807, Taiwan; ^2^Department of Medical Research, Kaohsiung Medical University Hospital, Kaohsiung 807, Taiwan; ^3^Department of Biological Sciences, National Sun Yat-Sen University, Kaohsiung 804, Taiwan; ^4^Department of Medical Science & Institute of Bioinformatics and Structural Biology, National Tsing Hua University, Hsinchu 300, Taiwan; ^5^Graduate Institute of Medicine, College of Medicine, Kaohsiung Medical University, Kaohsiung 807, Taiwan; ^6^Institute for Translational Research in Biomedicine, Kaohsiung Chang Gung Memorial Hospital, Kaohsiung 833, Taiwan; ^7^Department of Medical Laboratory Science and Biotechnology, Kaohsiung Medical University, Kaohsiung 807, Taiwan; ^8^Department of General Surgery, Changhua Christian Hospital, Changhua 500, Taiwan; ^9^School of Medicine, Kaohsiung Medical University, Kaohsiung 807, Taiwan; ^10^Center for Cancer Research, Kaohsiung Medical University, Kaohsiung 807, Taiwan; ^11^Drug Development and Value Creation Research Center, Kaohsiung Medical University, Kaohsiung 807, Taiwan; ^12^Division of General and Digestive Surgery, Department of Surgery, Kaohsiung Medical University Hospital, Kaohsiung 807, Taiwan; ^13^Department of Surgery, School of Medicine, College of Medicine, Kaohsiung Medical University, Kaohsiung 807, Taiwan

## Abstract

Lung cancer is a leading cause of cancer death worldwide, and non-small-cell lung cancer (NSCLC) accounts for 85% of lung cancer, which is highly metastatic, leading to the poor survival rate of patients. We recently reported that 4-[4-(4-hydroxyphenoxy)phenoxy]phenol (4-HPPP), a phenoxyphenol, exerts antihepatoma effects by inducing apoptosis and autophagy. In this study, we further examined the effect of 4-HPPP and its analogs on NSCLC cells. Colony formation assays showed that 4-HPPP exerts selective cytotoxicity against NSCLC H1299 cells; furthermore, the inhibitory effect of 4-HPPP on the proliferation and migration of NSCLC cells was validated using an *in vivo* zebrafish-based tumor xenograft assay. The flow cytometry-based dichlorofluorescein diacetate (DCF-DA) assays indicated that 4-HPPP caused an increase in reactive oxygen species (ROS) in NSCLC cells, and Western blot assays showed that the major ROS scavenging enzymes superoxide dismutases- (SODs-) 1/2 were upregulated, whereas peroxidase (PRX) was downregulated. Furthermore, 4-HPPP caused both aneuploidization and the accumulation of *γ*H2AX, a sensor of DNA damage, as well as the activation of double-strand break (DSB) markers, especially Ataxia-telangiectasia-mutated and Rad3-related (ATR) in NSCLC cells. Our present work suggests that the antiproliferative effects of 4-HPPP on lung cancer cells could be due to its phenoxyphenol structure, and 4-HPPP could be a candidate molecule for treating NSCLC by modulating ROS levels and lowering the threshold of polyploidy-specific cell death in the future.

## 1. Introduction

Lung cancer has a high incidence and is the leading cause of cancer-associated deaths in both males and females [1–3]. Non-small-cell lung carcinoma (NSCLC) is the most common type of lung cancer, accounting for 80% to 85% of lung cancer, and it shows a low proliferation rate and metastatic ability. Large-cell lung carcinoma accounts for 10% to 15% of NSCLC and is poorly differentiated [4, 5]. These tumors are large peripheral masses associated with early metastases. Surgery, radiotherapy, and chemotherapy are the main treatments for lung cancer. Although chemotherapy is the most common treatment for NSCLC [2–4, 6], NSCLC usually develops acquired resistance to chemotherapy and is associated with poor prognosis and low survival rates; therefore, continued efforts are still needed to overcome these difficulties [7, 8].

Aneuploidy is frequently observed in advanced cancer cells, and previous studies reported that at least 70% of common solid cancers are aneuploidy [9–11]. Cancer cells with aneuploidy may have enhanced proliferation and the ability to adapt to external stress or may be more chemoresistant [12]. A high rate of chromosomal missegregation has been reported to cause instability of chromosomes and poor prognosis in patients with diffuse large B cell lymphoma [13]. However, hyperaneuploidy beyond a certain level can be lethal or harmful to cancer cells [14, 15]. Therefore, elevating the rates of chromosome instability, such as aneuploidization or polyploidization, thereby inducing vulnerability of cancer cells to mitotic catastrophe [16] or apoptosis [14, 17], may be a promising strategy for treating cancer.

Phenoxyphenol derivatives have been reported to possess the capacity to prevent and treat colon and prostate cancers [18, 19]. Specifically, Parsai et al. reported that 4 phenoxyphenol derivatives exerted antimetastasis and anti-inflammatory activity by possibly binding to the active site of matrix metalloproteinase- (MMP-) 9 and cyclooxygenase (COX) 2 [20]. Additionally, we previously demonstrated that synthetic phenoxyphenol 4-[2356-tetrafluoro-4-(4-hydroxyphenoxy)phenoxy]phenol (TFPP) preconditioned NSCLC to respond to the anticancer agent camptothecin possibly by lowering the threshold for initiating apoptosis [21].

More recently, we identified that the synthetic phenoxyphenol 4-[4-(4-hydroxyphenoxy)phenoxy]phenol (4-HPPP) selectively killed hepatocellular carcinoma (HCC) cells and induced significant accumulation of *γ*H2AX, a DNA damage sensor [22], through modulating autophagy and inducing apoptosis. However, the effect of 4-HPPP on other cancer cells and its underlying mechanism remain unclear.

Given the potential anti-HCC activity of 4-HPPP, we further tested whether 4-HPPP exerts inhibitory effects on NSCLC using *in vitro* and *in vivo* zebrafish-based xenograft assays. Furthermore, the possible mechanisms by which 4-HPPP induced increased reactive oxygen species (ROS) and modulated the threshold of polyploidy-specific cell death of NSCLC are discussed.

## 2. Materials and Methods

### 2.1. Source of Diphenoxy Benzene Compounds

Four diphenoxy benzene compounds, including 4-HPPP, were purchased from the Enamine Ltd. (http://www.enamine.net, Kyivska region, Ukraine) chemical database (REAL Database). Four diphenoxy benzene compounds were freshly dissolved in DMSO at a concentration of 10 mM and stored at -20°C, and concentrations of 0.5, 1, 5, and 10 *μ*M were used to treat cells and zebrafish.

### 2.2. Cell Lines

The human NSCLC cell line H1299 was obtained from the American Type Culture Collection (ATCC, Manassas, VA, USA), and the human bronchial epithelium cell line BEAS-2B was kindly provided by Dr. Poling-Kuo (Kaohsiung Medical University, Taiwan). All the tested cells were maintained in Dulbecco's modified Eagle's medium (DMEM)/F-12 (3 : 2 ratio) and supplemented with 10% fetal bovine serum (FBS), 2 mM glutamine, and antibiotics (100 units/ml penicillin and 100 *μ*g/ml streptomycin) at 37°C with a humidified atmosphere of 5% CO_2_.

### 2.3. Reagents

The following compounds were obtained from Gibco BRL (Gaithersburg, MD, USA): DMEM, FBS, trypan blue, penicillin G, and streptomycin. Dimethyl sulfoxide (DMSO), paraformaldehyde (#P6148), ribonuclease A (RNase A, #R-4642), and propidium iodide (PI) were purchased from Sigma-Aldrich (St. Louis, MO, USA). An annexin V-fluorescein isothiocyanate (FITC) staining kit was purchased from Strong Biotech (#AVK050, Taipei, Taiwan). 4-[3-(4-iodophenyl)-2-(4-nitrophenyl)-2H-5-tetrazolio]-1,3-benzenedisulphonate (WST-1) was purchased from Takara Biomedicals (#MK400, Otsu, Japan). Antibodies against *γ*H2AX (#sc-101696), phosphorylated Akt (Ser^473^, #sc-7985-R), phosphor-ATR (#sc-109912), and phosphor-ATM (#sc-47739) proteins were purchased from Santa Cruz Biotechnology (Santa Cruz, CA, USA). Phosphor-Akt (Thr^450^, #3188-1) was purchased from Epitomics (Burlingame, CA, USA). Akt1 (#ab32505) was purchased from Abcam (Cambridge, UK). Bcl-2 (#GTX100064), peroxidase (PRX1) (#GTX101705), superoxide dismutase 1 (SOD1) (#GTX100659), SOD2 (#GTX116093), and glyceraldehyde-3-phosphate dehydrogenase (GAPDH) (#GTX627408) were purchased from GeneTex (Irvine, CA, USA). DNA-dependent protein kinase (DNA-PK) (#556456) was purchased from BD Pharmingen™ (San Jose, CA, USA). Horseradish peroxidase- (HRP-) conjugated secondary antibodies (#20102 for goat anti-mouse IgG and #20202 for goat anti-rabbit IgG) were purchased from Leadgene Biomedical Inc., Tainan, Taiwan. Fluorescein isothiocyanate- (FITC-) conjugated secondary antibodies (#GTX26816 for goat anti-mouse IgG and #GTX26798 for goat anti-rabbit IgG) were purchased from GeneTex.

### 2.4. Colony Formation Assay

A total of 1 × 10^2^ H1299 cells were seeded onto a 6-well plate and then incubated for 24 h. The cells were treated with four diphenoxy benzene compounds at different concentrations (0, 0.5, 1, 5, and 10 *μ*M). After 14 days of incubation, the cell colonies were fixed in glutaraldehyde and stained with crystal violet (1% *w*/*v*; Merck, #1408, Darmstadt, Germany) for 1 h. The diameter of the colonies was determined by Image-Pro Plus software (Media Cybernetics, Maryland, USA).

### 2.5. Cell Viability Assay

Cell viability was assessed by a WST-1 assay as described previously. Briefly, 1 × 10^3^ cells/well were seeded on a 96-well plate (tissue culture grade, flat bottom) in a final volume of 100 *μ*l/well culture medium in a humidified atmosphere (37°C, 5% CO_2_); after 24 h, the cells were treated with different concentrations of Akt-target compounds (0, 0.5, 1, 5, and 10 *μ*M) and cultured for 48 h. Next, 10 *μ*l/well WST-1 reagent was added to each well, and cells were incubated for 30 minutes in a humidified atmosphere (37°C, 5% CO_2_). The absorbance of the samples was measured at 450 nm against the background of a blank control using a microplate (ELISA) reader (Multiskan Ascent 354 microplate reader, Thermo Fisher Scientific, Rockford, IL, USA).

### 2.6. Cell Cycle Distribution Assay

A total of 2 × 10^5^ cells were seeded on a 12-well plate. After 24 h, different concentrations of Akt-targeting compounds (0, 0.5, 1, 5, and 10 *μ*M) were treated for 48, 72, and 96 h. The supernatant and cells were collected in a 1.5 ml tube, washed with PBS, fixed with 70% ethanol, and stored at -20°C for at least two hours. The ethanol was then removed, and the samples were washed with PBS and treated with Ribonuclease (RNase) A in 100 *μ*l PBS (40 *μ*g/ml) for 30 minutes. The RNase A was removed and washed with PBS, and the samples were treated with 20 *μ*g/ml PI in PBS. The cells were analyzed by flow cytometry (FACSCalibur, BD Biosciences, San Jose, CA, USA) using FlowJo 7.5.5 software (Tree Star, Inc., San Carlos, CA).

### 2.7. Assessment of Apoptosis

To examine the apoptosis-inducing potential of 4-HPPP in H1299 cells, annexin V/PI double staining was performed to detect the externalization of phosphatidylserine (PS). In brief, 2 × 10^5^ cells were seeded onto 12-well plates and treated with or without 4-HPPP for 48 h and 72 h. Subsequently, the cells were harvested and stained with the annexin V/PI kit (Strong Biotech) according to the manufacturer's instructions. Cells were analyzed by flow cytometry (FACSCalibur) using FlowJo v7.5.5 software (Tree Star, Inc.).

### 2.8. Intracellular ROS Detection Assay

The amount of endogenous H_2_O_2_ was detected through an oxidation-sensitive fluorescence dye 2′,7′-dichlorofluorescein diacetate (DCF-DA). DCF-DA is able to penetrate the cell membrane. While entering the cell, it is cleaved and oxidized by H_2_O_2_ in cells, forming a DCF product with green fluorescence at wavelengths between 488 and 530 nm [23–26]. Briefly, 2 × 10^5^ cells were seeded on a 12-well plate and treated with or without 4-HPPP for 24 h and 48 h. Afterward, cells were treated with different concentrations of 4-HPPP and its analogs (from 0.5 to 10 *μ*M) for 24 h and 48 h. The supernatants were removed and washed with PBS, followed by the addition of 0.1 *μ*M DCF-DA (2′,7′-dichlorofluorescein diacetate) (Sigma-Aldrich, St. Louis, Missouri, USA) to the 12-well plates for 30 minutes at 37°C. Then, the DCF-DA was removed, and the cells were washed with PBS. Cells were collected in 1.5 ml tubes and analyzed by flow cytometry (FACSCalibur) using FlowJo v7.5.5.

### 2.9. Cytometric Assessment of Protein Phosphorylation

DNA damage was analyzed in H1299 cells after 4-HPPP treatment by a flow cytometry-based assay to detect the activation of *γ*H2AX, phosphor-ATM, phosphor-ATR, and DNA-PK in cells [27]. A total of 2 × 10^5^ cells were seeded on a 12-well plate and treated with or without 4-HPPP. After 24 h, cells were treated with the indicated concentrations (0, 0.5, 1, 5, and 10 *μ*M) of 4-HPPP. Cells were harvested and washed with PBS and fixed with 70% ethanol at -20°C. The alcohol was removed, and cells were washed with BSA-T-PBS (1% BSA, 0.5% Triton in PBS), followed by the addition of *γ*H2AX, phosphor-ATM, phosphor-ATR, or DNA-PK primary antibodies. The samples were then washed with BSA-T-PBS, and the fluorescent secondary antibody was added. Finally, the samples were washed with BSA-T-PBS and treated with 20 *μ*g/ml PI in BSA-T-PBS. The cells were analyzed by flow cytometry (FACSCalibur, BD Biosciences, San Jose, CA, USA) using FlowJo 7.5.5 software (Tree Star, Inc.).

### 2.10. Western Blot Analysis

Western blotting assays were conducted as described previously [28]. Briefly, the cells were harvested and lysed. Lysates were centrifuged, and the protein lysate concentrations were determined using a Pierce™ bicinchoninic acid (BCA) protein assay kit (#23225, Thermo Scientific Pierce Protein Research, Rockford, IL, USA). Equal amounts of protein (20 *μ*g) were separated by SDS-polyacrylamide gel electrophoresis (SDS-PAGE) and then electrotransferred to a 0.2 *μ*m polyvinylidene difluoride (PVDF) membrane (Pall, FL, USA). The PVDF membrane was blocked with 5% nonfat milk and sequentially incubated with primary and secondary antibodies against specific proteins. The signal intensities were detected using an enhanced chemiluminescence (ECL) detection kit (Amersham, Piscataway, NJ, USA).

### 2.11. Cellular Motility Assessment

The cellular motility was determined using Boyden's transwell assay. Briefly, H1299 cells were seeded on a transwell insert with 8 *μ*m pore polycarbonate filters (Greiner Bio-One, Frickenhausen, Germany), and the lower well contained medium with 10% FBS without or with the indicated concentrations of 4-HPPP for 18 h. Cells on the bottom surface of the filters were paraformaldehyde-fixed and Giemsa-stained; then, all cells were counted under a microscope (Nikon Eclipse TE2000-U, Tokyo, Japan). The experiment was performed in triplicate, and the results of three independent experiments are presented as the mean ± SD.

### 2.12. Zebrafish Husbandry

Adult *Tg*(Fli1:GFP)^y1^ zebrafish were provided by the Zebrafish International Resource Center (ZIRC), Taiwan Zebrafish Core at National Health Institutes and Tsing Hua University (TZeTH), Hsinchu, Taiwan (http://icob.sinica.edu.tw/tzcas/fishlineszeth.html). Zebrafish were maintained in a 14 h light/10 h dark cycle at 28°C.

### 2.13. Toxicity in Zebrafish

Before the xenograft assay, the toxicity of 4-HPPP in zebrafish was tested by exposing zebrafish larvae at 2 days postfertilization (dpf) to 4-HPPP for 48 h. As toxicological endpoints, the images of abnormal larvae were recorded and calculated after 24 and 48 h of exposure.

### 2.14. Zebrafish Xenograft

To further validate the antilung cancer effect of 4-HPPP, we transfected the plasmid pDsRed-Express-C1 (Clontech, Mountain View, CA, USA) into human tumor cells for tracking the xenografted cells by fluorescence microscopy. The zebrafish-based xenograft assay was performed according to our previous study with minor modifications [29]. Briefly, 48 h postfertilization (hpf) zebrafish larvae were anesthetized with 0.01% tricaine and transplanted with approximately 50 lung cancer cells per larva by microinjection. The larvae were incubated in water with 4-HPPP for 24 h and 48 h postinjection. Afterward, images were captured with an inverted fluorescence microscope (Nikon Eclipse TE2000-U, Tokyo, Japan).

### 2.15. Statistical Analysis

Differences between 4-HPPP- and DMSO- (as vehicle control) treated cells were analyzed in at least triplicate experiments. The differences were analyzed by one-way analysis of variance (ANOVA) with *p* < 0.05 considered significant. For the *in vivo* zebrafish xenograft assay, the metastasis potential was assessed by Fisher's exact test according to the previous study of Tang et al. [30].

## 3. Results

### 3.1. 4-HPPP Reduces Colony Formation Capacity in NSCLC

**Figure 1:**
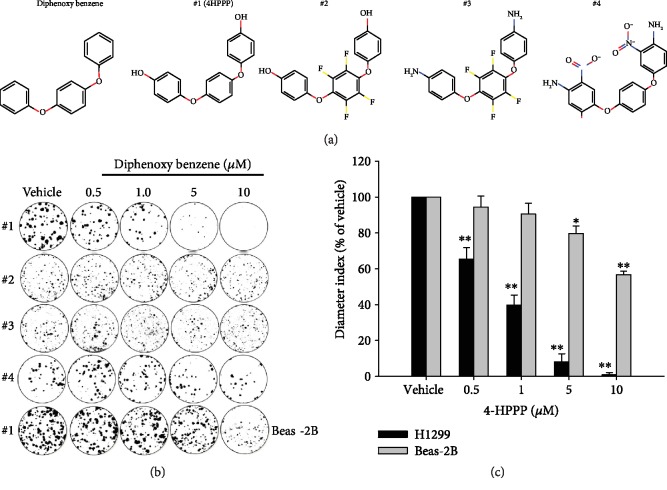
The inhibitory effect of compounds on the long-term proliferation of NSCLC cells. NSCLC H1299 cells and BEAS-2B human bronchial epithelial cells were treated with the indicated concentrations (from 0.5 to 10 *μ*M) of tested compounds for 14 days. Afterward, the cells were paraformaldehyde-fixed and stained with crystal violet. (a) Chemical structures of 4-HPPP and its structural analogs. (b) Representative results of the colony formation of H1299 and BEAS-2B cells following compound treatment. (c) The quantitative results of (b) were statistically analyzed with one-way ANOVA. ^∗^*p* < 0.05; ^∗∗^*p* < 0.001. Vehicle control vs. 4-HPPP treatments. #1: 4-HPPP; #2: 4-[2356-tetrafluoro-4-(4-hydroxyphenoxy)phenoxy]phenol; #3: 4-[4-(4-aminophenoxy)-2356-tetrafluorophenoxy]aniline; #4: 4-[4-(4-amino-3-nitrophenoxy)phenoxy]-2-nitroaniline.

Because 4-HPPP also belongs to the diphenoxy benzene family, we were interested whether other diphenoxy benzene compounds with different modifications could have cytotoxicity effects similar to those of 4-HPPP against cancer cells; the diphenoxy benzene compounds were obtained from the chemical company Enamine Ltd. (https://enamine.net/) and predicted to have Akt-targeting effects according to the bioinformatics approaches of Enamine Ltd. (Figure 1(a)). The results of the WST-1 assay showed that 4-HPPP moderately inhibited cell viability, but not in a dose-dependent manner ([Supplementary-material supplementary-material-1]). We then examined whether 4-HPPP reduced the clonogenicity of NSCLC cells, and a colony formation assay was conducted (Figure 1(b)). Interestingly, the results showed that 4-HPPP dramatically reduced the clonogenicity capacity of H1299 cells in a dose-dependent manner, suggesting a long-term inhibitory effect of 4-HPPP on the clonogenic capacity of NSCLC cells compared to that of other diphenoxy benzene compounds. Importantly, only a slight reduction in colony formation of 4-HPPP-treated normal lung bronchia BEAS-2B cells was observed (Figures 1(b) and 1(c)) compared with NSCLC cells, showing that the inhibitory effects of 4-HPPP were selective to NSCLC cells rather than normal lung cells.

### 3.2. 4-HPPP Induces Apoptosis in NSCLC Cells

**Figure 2:**
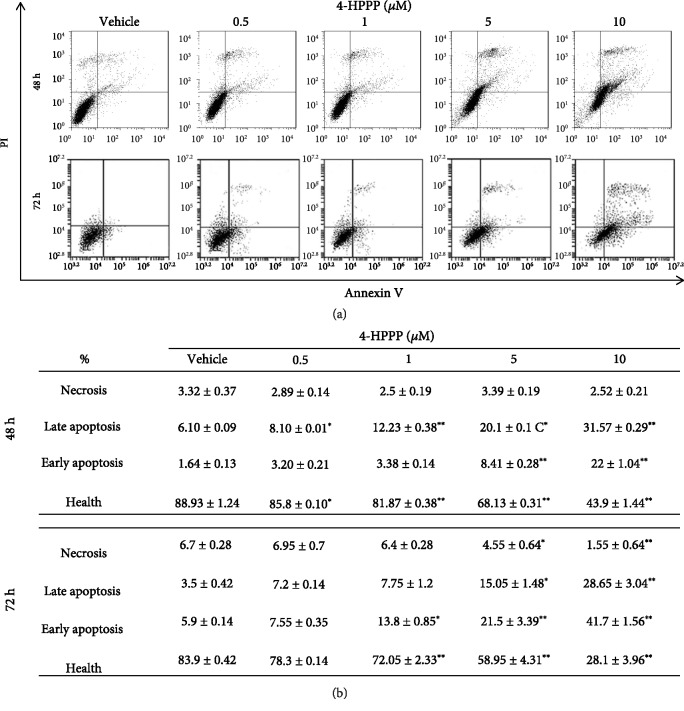
4-HPPP induces apoptosis of NSCLC H1299 cells. The apoptosis induced by 4-HPPP was assessed using flow cytometry-based annexin V-PI staining. (a) H1299 cells were treated with 4-HPPP for the indicated time course. (b) The quantitative results of (a). *p* < 0.05 (vehicle vs. 4-HPPP treatment) was considered statistically significant. ^∗^*p* < 0.05; ^∗∗^*p* < 0.001.

**Figure 3:**
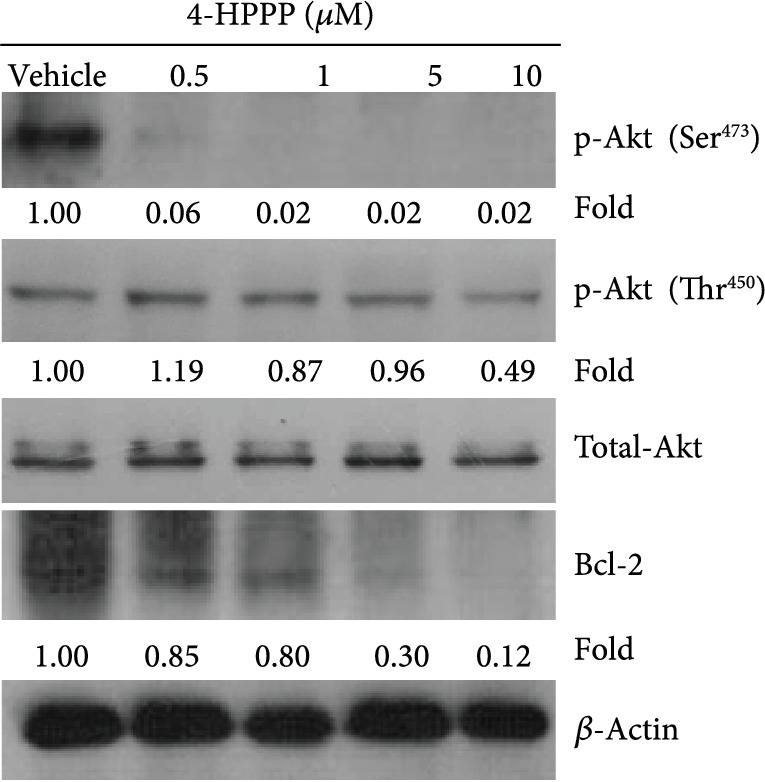
The effect of 4-HPPP on Akt phosphorylation changes in NSCLC cells. The phosphorylation changes at serine^473^ and threonine^450^ of Akt along with the prosurvival factor Bcl-2 were assessed using the Western blotting assay. *β*-Actin was used as an internal control to ensure equal loading.

As shown in Figures 2(a) and 2(b), the apoptosis of H1299 cells significantly increased at treatment concentrations of 5 and 10 *μ*M. In addition, the Western blot results revealed that after 4-HPPP treatment, both the phosphorylation of prosurvival p-Akt (Ser^473^ and Thr^450^) and its downstream protein Bcl-2 were downregulated, whereas there were no significant changes in total Akt protein (Figure 3), suggesting that 4-HPPP regulates the activity of Akt rather than its protein level.

### 3.3. 4-HPPP Induces Polyploidy in NSCLC Cell Lines

**Figure 4:**
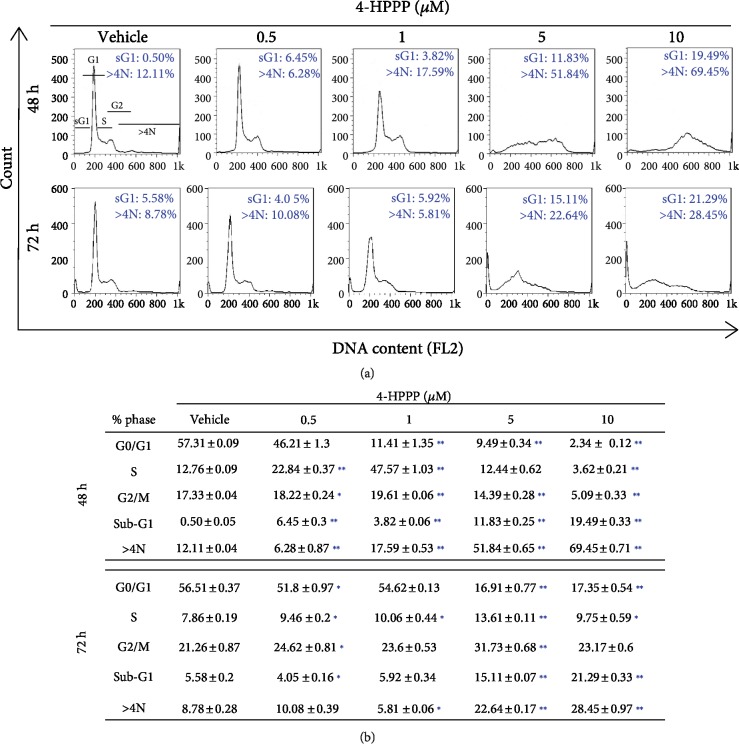
The effect of 4-HPPP on cell cycle progression. (a) The accumulation of sub-G_1_ and aneuploidy (N>4N) in NSCLC cells. H1299 cells were seeded and treated with different concentrations of 4-HPPP for 48 and 72 h. The treated cells were 70% ethanol-fixed and PI-stained and then subjected to a cell cycle distribution analysis by flow cytometry. Changes in cell cycle progression, the sub-G_1_ population, and aneuploidy following 4-HPPP treatment. (b) Quantitative analysis of cell cycle progression. ^∗^*p* < 0.05; ^∗∗^*p* < 0.001.

To examine whether 4-HPPP induced NSCLC cell cycle disturbances, H1299 cells were treated with different concentrations of 4-HPPP (from 0.5 to 10 *μ*M) for 48 and 72 h. The cells were then stained with PI to assess cell cycle distribution. The results showed that treatment with higher concentrations (5 and 10 *μ*M) of 4-HPPP for 48 h significantly increased polyploidy or aneuploidy. After treatment for 72 h, the population of sub-G_1_, a hallmark of apoptosis, dramatically accumulated, suggesting that the induction of apoptosis by 4-HPPP is both dose- and time-dependent (Figures 4(a) and 4(b)).

### 3.4. 4-HPPP Induces DNA Damage of H1299

**Figure 5:**
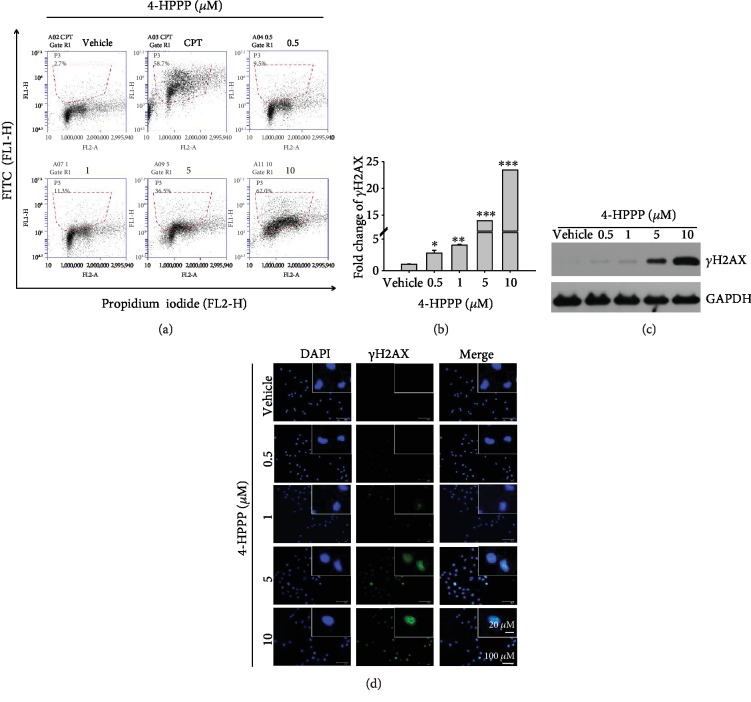
Assessment of DNA damage induced by 4-HPPP. (a) H1299 cells were administered the indicated concentrations of 4-HPPP for 48 h. Afterward, 4-HPPP-induced DNA damage was detected using a flow cytometry-based *γ*H2AX detection assay. The green fluorescence of FITC (FL1) indicates the *γ*H2AX-positive population (R1 region). The data showed that 4-HPPP increased the phosphorylation of *γ*H2AX, a marker of DNA damage, in a dose-responsive manner. (b) Quantification analysis of (a). *γ*H2AX was observed in H1299 cells following 4-HPPP treatment at concentrations from 0.5 to 10 *μ*M by the Western blotting assay (c) and immunofluorescence assay (d). The data are presented as the mean ± SD. ^∗^*p* < 0.05; ^∗∗^*p* < 0.005; ^∗∗∗^*p* < 0.001. Scale bar: 100 *μ*m.

DNA damage is the major cause of aneuploidy or polyploidy in cancer cells [31, 32]. To determine whether 4-HPPP caused aneuploidy or polyploidy or triggered apoptosis in NSCLC cells, we conducted flow cytometry-based immunostaining and Western blotting to detect changes in the DNA damage sensor *γ*H2AX (the phosphorylated form of the histone protein H2AX) [33]. The results showed that the fold of *γ*H2AX-activated cells was increased by increasing the 4-HPPP concentration (Figures 5(a) and 5(b)). Consistently, the level of *γ*H2AX was increased in a dose-dependent manner at 48 h post-4-HPPP treatment (Figure 5(c)). We also evaluated the distributions of foci of *γ*H2AX in 4-HPPP-treated H1299 cells using immunofluorescence staining. Significant accumulation of *γ*H2AX foci was detected in cells treated with 5 and 10 *μ*M 4-HPPP (Figure 5(d)), indicating that 4-HPPP induced DNA damage, especially DNA double-strand breaks (DSBs), in H1299 cells.

**Figure 6:**
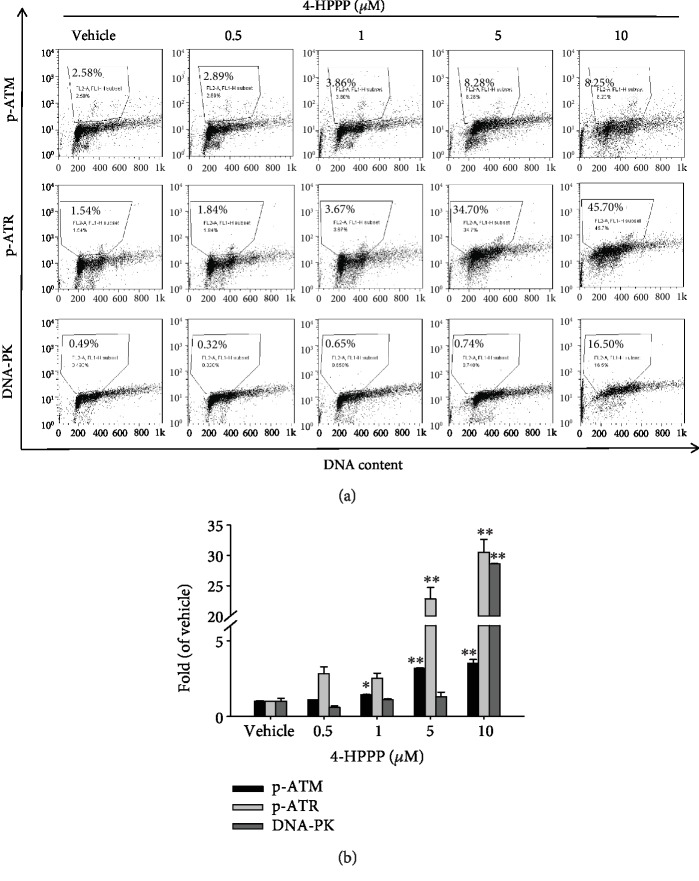
4-HPPP-induced activation of DNA damage markers in NSCLC cells. (a) H1299 cells were seeded and treated with 4-HPPP for 48 h. Afterward, the DNA damage induced by 4-HPPP was assessed by flow cytometry. The markers of DNA damage, including phosphor-ATR, phosphor-ATM, and DNA-PK, were determined. The results showed that 4-HPPP increased the phosphorylation of ATR and ATM and the protein level of DNA-PK in a dose-responsive manner. (b) The quantitative results of (a). ^∗^*p* < 0.05; ^∗∗^*p* < 0.001.

To study whether markers of DNA damage were activated in 4-HPPP-treated cells, phosphor-ATM, phosphor-ATR, and DNA-PK were detected by flow cytometry. As shown in Figures 6(a) and 6(b), significantly more phosphor-ATR-positive cells were found than both ATM- and DNA-PK-positive cells, which revealed that 4-HPPP causes DNA damage, leading to ATR activation.

### 3.5. 4-HPPP Increased Hydrogen Peroxide Production

**Figure 7:**
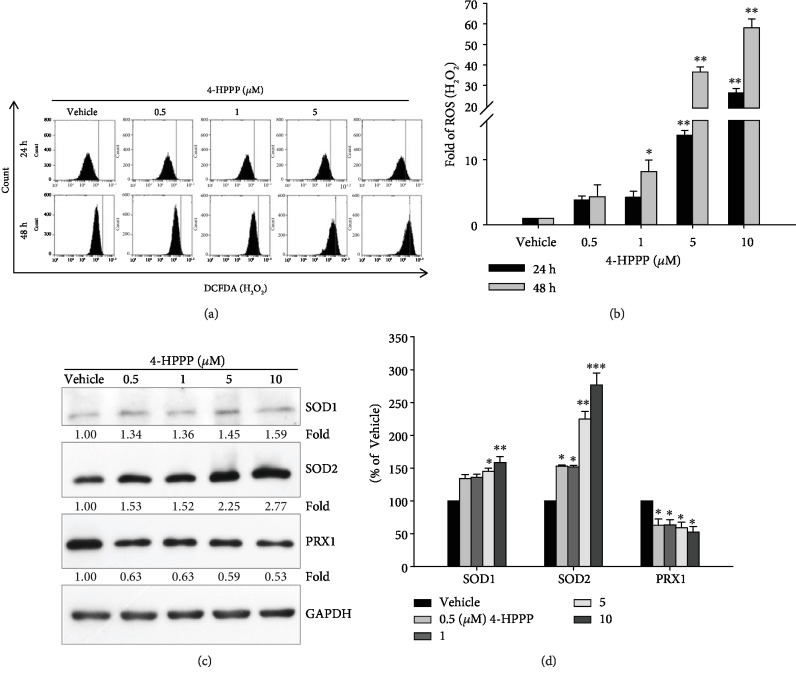
4-HPPP-induced changes in endogenous ROS and antioxidants in NSCLC cells. (a) H1299 cells were treated with the indicated concentrations of 4-HPPP for 24 h and 48 h. Afterward, intracellular levels of ROS were measured by the flow cytometry-based DCF-DA assay described in Materials and Methods. (b) Quantitative analysis of (a). (c) Changes in endogenous antioxidants SOD1, SOD2, and PRX1 in H1299 cells following 4-HPPP treatment by Western blotting. (d) Quantitative analysis of (c). ^∗^*p* < 0.05; ^∗∗^*p* < 0.01; ^∗∗∗^*p* < 0.001.

To determine whether 4-HPPP induces apoptosis through ROS, we detected intracellular hydrogen peroxide (H_2_O_2_), one of the major types of intracellular ROS, using flow cytometer-based DCF-DA staining. The results showed that 4-HPPP caused a dose-dependent increase in H_2_O_2_ (Figures 7(a) and 7(b)). Furthermore, Western blotting showed that the protein level of SOD2 was increased; in contrast, the peroxidase PRX1 was significantly decreased in a dose-dependent manner following 4-HPPP treatment (Figures 7(c) and 7(d)).

### 3.6. 4-HPPP Attenuates the Motility of NSCLC Cells

**Figure 8:**
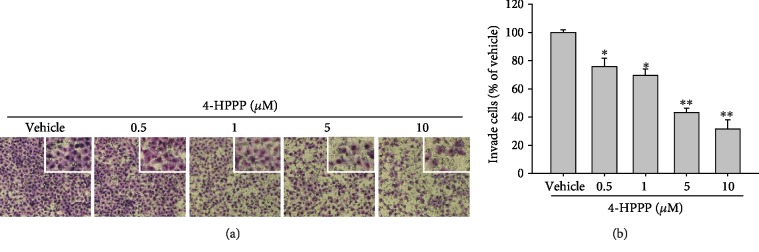
The effect of 4-HPPP on the motility of NSCLC cells. (a) H1299 cells were treated with the indicated concentrations of 4-HPPP for 18 h. Afterward, the cells were stained with 0.1% *w*/*v* Giemsa. (b) Quantitative analysis of (a). ^∗^*p* < 0.05 and ^∗∗^*p* < 0.01 for 4-HPPP treatments against vehicle control, respectively.

Figures 8(a) and 8(b) reveal that the motility of H1299 cells treated with the indicated concentrations of 4-HPPP at 0, 0.5, 1, 5, and 10 *μ*M was 100 ± 2.00, 75.85 ± 5.99, 69.48 ± 4.58, 43.22 ± 3.07%, and 31.48 ± 6.54% (*n* = 3), respectively, indicating that 4-HPPP attenuates motility, an index of metastasis of cancer in H1299 cells.

### 3.7. 4-HPPP Inhibits H1299 Cell Proliferation and Migration in Zebrafish Xenografts

**Figure 9:**
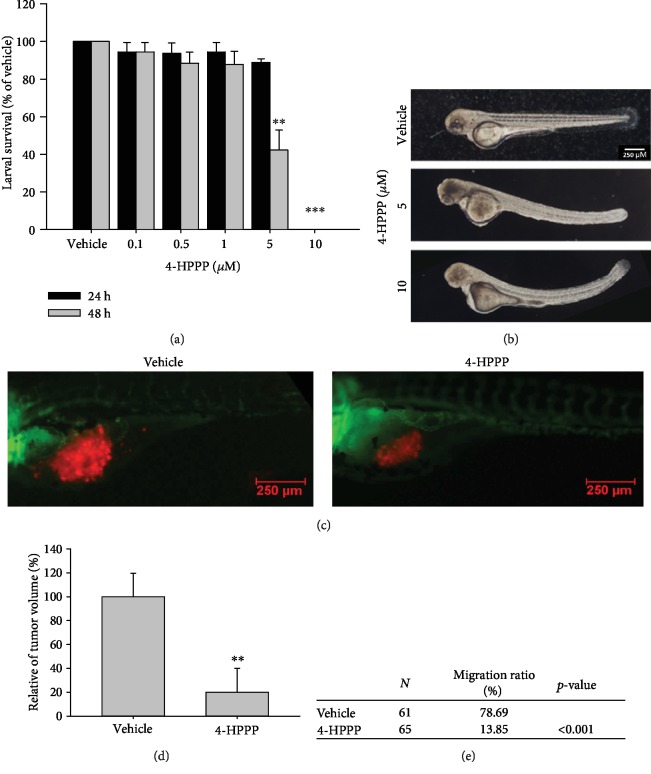
Effect of 4-HPPP on zebrafish-based xenografted NSCLC cells. (a) Survival rate of zebrafish larvae following 4-HPPP exposure. (b) The results showed that 5 *μ*M and 10 *μ*M 4-HPPP caused body axis bending and edema in zebrafish larvae, indicating that a high concentration of 4-HPPP could cause deformation and toxicity toward zebrafish larvae. (c) The motility of xenografted H1299 cells in the yolk sac of zebrafish larvae. The intensity of red fluorescence is proportional to the xenograft tumor size. For each group, the sample size of larvae (*N*) > 60. (d, e) Quantitative analysis of (c). The data are presented as the mean ± S.D. ^∗∗^*p* < 0.05 and ^∗∗∗^*p* < 0.001 against vehicle control. (e) The statistical analysis of the migration ability of xenografted H1299 cells using Fisher's exact test.

Before evaluating the anticancer activity of 4-HPPP, we examined whether 4-HPPP causes side effects in zebrafish. The results showed that the survival rates of zebrafish larvae did not change below 1 *μ*M 4-HPPP. However, the survival rate was only 42.2% after treatment with 5 *μ*M 4-HPPP for 48 h (Figure 9(a)). The higher concentrations of 5 *μ*M and 10 *μ*M 4-HPPP caused slight bending of the body axes and edema in the zebrafish larvae (Figure 9(b)), indicating that a high concentration of 4-HPPP induced moderate side effects in zebrafish.

To further examine the anticancer effect of 4-HPPP *in vivo*, we generated a lung cancer xenograft zebrafish model by microinjection of fluorescent H1299 cells. Red fluorescent reporter H1299 cells were obtained by transfection with the pDsRed-Express-C1 vector (Figure 9(c)). Fluorescent images at 48 h posttreatment revealed a reduction in tumor volume and migration in 4-HPPP-treated xenografted H1299 cells (Figures 9(d) and 9(e)).

## 4. Discussion

It has been reported that most NSCLC cells with mutations and deletions of the tumor suppressor p53 are insensitive to anticancer drugs such as cisplatin [34]. In contrast, Akt signaling has been reported to be frequently overexpressed or dysregulated in NSCLC, resulting in the activation of the PI3K/Akt pathway, which inhibits apoptosis and is correlated with radioresistance [35]. Furthermore, Akt activity increases as cells progress through the G_2_/M phase [36]. Given that both p53 mutation and Akt overexpression are closely correlated with the chemoresistance and prognosis of NSCLC patients, Akt may play a pivotal role in NSCLC pathogenesis and, thus, represents an ideal target for therapeutic intervention.

We previously demonstrated that the synthetic phenoxyphenol 4-[4-(4-hydroxyphenoxy)phenoxy]phenol (4-HPPP) inhibits cellular proliferation and induces apoptosis in hepatocellular carcinoma cells [22]. In this study, we used H1299 cells, which carry null-*p*53 and constitutively active Akt, as a cell model to further examine the effect of 4-HPPP on NSCLC cells. First, we examined the effects of 4-HPPP and three structurally similar compounds (diphenoxy benzenes) on the viability and clonogenicity of H1299 cells. The results showed that among the tested compounds, 4-HPPP moderately inhibited the viability of NSCLC H1299 cells at 24 h and 48 h ([Supplementary-material supplementary-material-1] and [Supplementary-material supplementary-material-1]). However, 4-HPPP had the most potential for inhibiting the colony formation of NSCLC cells (Figure 1), suggesting the long-term inhibitory effect of 4-HPPP on NSCLC cell clonogenicity. Importantly, 4-HPPP also preferentially inhibited colony formation in H1299 cells but not in BEAS-2B normal lung cells. The results suggested that the selective inhibitory effect of 4-HPPP may be a promising treatment in the future.

Our previous study showed that 4-HPPP potentially inhibits the phosphorylation of Akt at Ser^473^ and Thr^450^ in HCC Huh7 cells. Consistently, our results showed that 4-HPPP inhibited the activation of Akt in NSCLC H1299 cells (Figure 3). These observations confirmed that 4-HPPP could be a specific inhibitor of Akt, which may benefit the development of Akt-targeting drugs for treating cancer in the future.

We further assessed the cell cycle distribution after 4-HPPP treatment. The results revealed that 4-HPPP induced hyperaneuploidization of NSCLC cells in a dose-dependent manner (Figure 4). A recent study proposed that the induction of hyperaneuploidization or hyperpolyploidization can be considered beneficial for inducing the apoptosis of cancer cells that carry aneuploidy or polyploidy. For example, colorectal cancer cells with high polyploidy exhibited a positive response to cotreatment with irinotecan combined with 5-fluorouracil in a clinical trial [37].

We noted that 4-HPPP treatment caused S-phase cell accumulation at the 1 *μ*M concentration, but this accumulation was decreased at 5 and 10 *μ*M; moreover, the level of aneuploidy increased in a dose-dependent manner, suggesting the correlation of S-phase arrest and high-grade aneuploidization/polyploidization. Liu et al.'s work reported that a topoisomerase I inhibitor, a synthetic analog of camptothecin TCH-1030, caused S-phase arrest and finally resulted in polyploidization and sequentially apoptosis in a panel of breast cancer cell lines [38]. Their results suggested that TCH-1030 induces DNA damage and S-phase cell cycle arrest by impairing mitosis and cytokinesis, eventually leading to polyploidization (>4N) and apoptosis.

Another study also reported the correlation of sub-G_1_ cell cycle arrest and hyperpolyploidization. For example, Karna et al.'s work suggested that the novel microtubule-modulating noscapinoid EM011 dysregulates cell division and the asymmetric distribution of DNA, resulting in both high-grade polyploidy and sub-G_1_ cell accumulation, eventually causing tumor suppression and promoting death in breast cancer MCF-7 cells [39]. Similarly, our previous work revealed the antimicrotubule effect of 4-HPPP in HCC cells [22]; we noted that at 48 h, the 5 *μ*M and 10 *μ*M doses suddenly increased the number of cells in the sub-G_1_ phase, and aneuploidy increased simultaneously, suggesting that the accumulation of sub-G_1_ cells, a marker of apoptosis, might be caused by 4-HPPP-induced excess aneuploidization in H1299 cells. We further confirmed that 4-HPPP induces apoptosis in H1299 cells using annexin V staining (Figure 2). Thus, the above observations provide information about the mechanism underlying the S-phase cell arrest and sub-G_1_ cell increase induced by 4-HPPP treatment.

Earlier studies have indicated that polyploidy might be induced in cancer cells by severe DNA damage, especially DSBs [5, 6, 31, 40]. When cells are exposed to radiation or chemicals that cause DNA damage, DSBs are induced, and a subunit of histone, H2AX, is quickly phosphorylated at Ser^139^ to form *γ*H2AX. Therefore, *γ*H2AX is considered a marker of DNA damage [8, 33]. Our previous work demonstrated that 4-HPPP induces H2AX accumulation in HCC Huh7 and Ha22T cells [22]. In this study, to determine whether 4-HPPP causes polyploidization by inducing DNA damage in NSCLC cells, we analyzed *γ*H2AX activation following 4-HPPP treatment (Figures 5(a) and 5(b)). The level of polyploidy induced by 4-HPPP was positively correlated with the activation of *γ*H2AX in H1299 cells (Figures 5(c) and 5(d)). We also checked the activation of major DNA repair factors, including phosphor-ATM, phosphor-ATR, and DNA-PK, and the level of ATR activation was much higher than that of the other two factors following 4-HPPP treatment, suggesting that ATR may play a major role in the signaling of DNA damage (Figure 6).

Moderate levels of intracellular ROS, including hydroxyl radicals (^·^OH), superoxide (O_2_^−^), and H_2_O_2_, are essential for the proliferation and survival of cells [41, 42]. ROS play an important role in signal transduction related to survival, proliferation, angiogenesis, and metastasis in cancer cells [43–46]. However, excess ROS usually cause severe damage or apoptosis of cells, and upregulating endogenous ROS has been considered to be a promising strategy for eliminating cancer cells [47–49]. Therefore, our results also demonstrated that 4-HPPP significantly increased endogenous ROS through modulating the expression of antioxidant enzymes such as SOD2 and peroxidase (Figures 7(a) and 7(b)).

Zebrafish (*Danio rerio*) is an excellent model for cancer research and drug discovery because of its rapid development and larval transparency [50–52]. Zebrafish xenografts provide a unique opportunity to monitor the tumor-induced angiogenesis, growth, and invasiveness of xenografted tumor cells. In addition, zebrafish are also a useful tool for evaluating the effects of drugs and their side effect [22, 28, 53]. First, we evaluated the toxicity of 4-HPPP to the larvae of zebrafish; the survival rates of zebrafish larvae did not change at low concentrations of 4-HPPP (Figure 9). However, the results showed that a high concentration of 4-HPPP caused the zebrafish larval body axes to bend and develop edema, indicating that a high concentration of 4-HPPP could cause unfavorable side effects in zebrafish (Figure 9(a)); all the zebrafish larvae died at the highest concentration of 4-HPPP treatment.

The results of the *in vivo* zebrafish-based xenograft assay showed that both the tumor mass and cellular migration of xenografted H1299 cells were significantly attenuated following 4-HPPP treatment (Figures 9(c)–9(e)), which is consistent with the *in vitro* results.

Accordingly, we thus suggest that 4-HPPP, a phenoxyphenol-based compound, could lead to DNA damage accumulation and increased ROS levels, resulting in *γ*H2AX and ATR activation to induce excess aneuploidization/polyploidization, ultimately causing cell apoptosis. 4-HPPP, which also exhibits Akt-targeting properties, inhibited the activity of the phosphorylated sites of Akt, Ser^473^and Thr^450^, which are involved in both NSCLC cell proliferation and metastasis.

## 5. Conclusions

**Figure 10:**
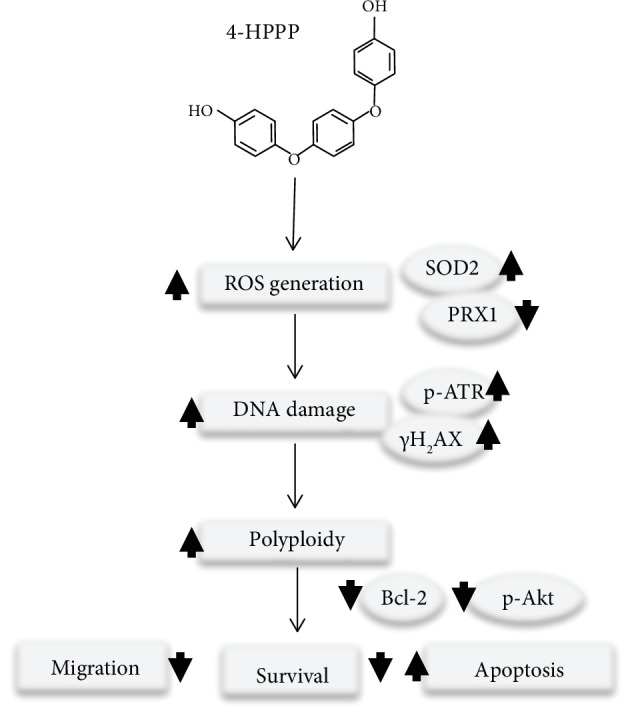
A proposed mechanism of 4-HPPP-induced antiproliferation and apoptosis in NSCLC cells through modulating the signaling of endogenous antioxidant systems, such as SOD2 and PRX1, causing DNA damage and high-grade aneuploidy. Eventually, the accumulation of DNA damage and hyperaneuploidization induce apoptosis in NSCLC cells.

Our study demonstrated that 4-HPPP selectively inhibits the growth of NSCLC H1299 cells. We also confirmed that 4-HPPP significantly inhibits the activation of Akt but increases the levels of ROS, DNA damage, and hyperpolyploidy, finally inducing apoptosis in H1299 cells (Figure 10). In addition to its antiproliferative effects, 4-HPPP inhibits migration, a marker of metastasis in NSCLC cells; therefore, for the first time, we demonstrated the antimigration effect of 4-HPPP, which may benefit the development of antilung metastasis therapies. The potent inhibitory effect of 4-HPPP on the proliferation and metastasis of H1299 cells was validated by an *in vivo* zebrafish-based xenograft assay. However, we also noted that a higher concentration of 4-HPPP caused body axis deformation and edema in zebrafish larvae. The structure of 4-HPPP could be further modified and optimized to reduce its side effects for further application.

## Data Availability

No data were used to support this study.

## Supplementary Materials

Supplementary Materials The inhibitory effect of 4-HPPP and its analogs on the short-term proliferation of NSCLC cells. H1299 cells were seeded and treated with the indicated concentrations of 4-HPPP or one of its analogs for 24 h (a) and 48 h (b). #1: 4-HPPP; #2: 4-[2356-tetrafluoro-4-(4-hydroxyphenoxy)phenoxy]phenol; #3: 4-[4-(4-aminophenoxy)-2356-tetrafluorophenoxy]aniline; and #4: 452 4-[4-(4-amino-3-nitrophenoxy)phenoxy]-2-nitroaniline. ^∗^*p* < 0.05 and ^&^*p* < 0.001, 4-HPPP compound treatments against vehicle control.
