# Supplementary material for: The Phenoxyphenol Compound 4-HPPP Selectively Induces Antiproliferation Effects and Apoptosis in Human Lung Cancer Cells through Aneupolyploidization and ATR DNA Repair Signaling
Source: Oxid Med Cell Longev. 2020 Jan 7;2020:5167292. doi: 10.1155/2020/5167292 (PMC7024103; doi:10.1155/2020/5167292)
Supplement: Supplementary Materials — The inhibitory effect of 4-HPPP and its analogs on the short-term proliferation of NSCLC cells. H1299 cells were seeded and treated with the indicated concentrations of 4-HPPP or one of its analogs for 24 h (a) and 48 h (b). #1: 4-HPPP; #2: 4-[2356-tetrafluoro-4-(4-hydroxyphenoxy)phenoxy]phenol; #3: 4-[4-(4-aminophenoxy)-2356-tetrafluorophenoxy]aniline; and #4: 452 4-[4-(4-amino-3-nitrophenoxy)phenoxy]-2-nitroaniline. ∗p < 0.05 and &p < 0.001, 4-HPPP compound treatments against vehicle control. [file 5167292.f1.pdf]

Supplementary Figure:

Figure S1

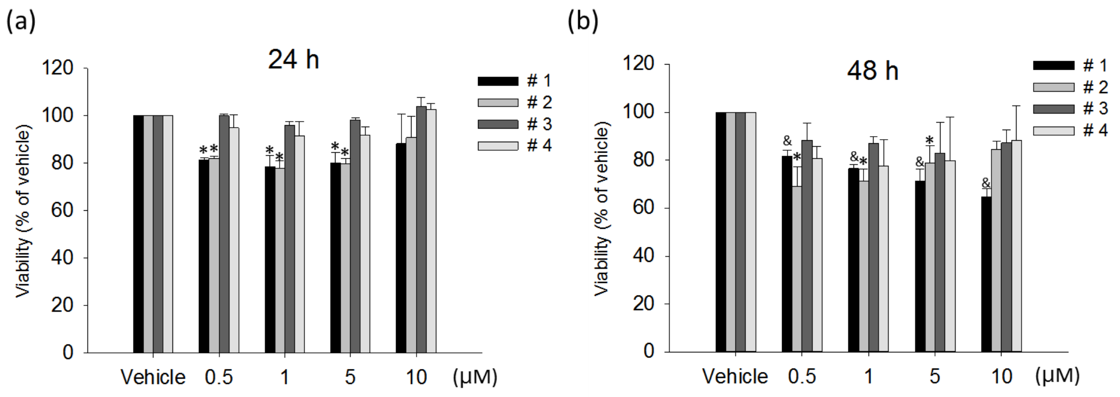

Figure S1: The inhibitory effect of 4-HPPP and its analogues on the short-term proliferation of NSCLC cells. H1299 cells were seeded and treated with the indicated concentrations of 4-HPPP or one of its analogues for 24 h (a) and 48 h (b). #1: 4-HPPP; #2: 4-[2356-tetrafluoro-4-(4-hydroxyphenoxy)phenoxy]phenol; #3: 4-[4-(4-aminophenoxy)-2356-tetrafluorophenoxy]aniline; #4: 4-[4-(4-amino-3-nitrophenoxy)phenoxy]-2-nitroaniline. $^{*}p < 0.05$ and $^{\&}p < 0.001$ 4-HPPP compound treatments against vehicle control.
